# A Phase 2 Clinical Trial of AR1004 in Patients with Mild Cognitive Impairment

**DOI:** 10.1002/alz.095652

**Published:** 2025-01-09

**Authors:** Fred Kim, Tianyang Xi, Jai Jun Choung, Eunjung Jeon, Gesun Choi

**Affiliations:** ^1^ AriBio Co., Ltd., San Diego, CA USA; ^2^ AriBio Co., Ltd., Seongnam Korea, Republic of (South)

## Abstract

**Background:**

Mild Cognitive Impairment (MCI) is a condition in which an individual shows declines in memory or mental function beyond the normal range for their age. Symptoms are not as severe as those with dementia, and people with MCI are able to carry out daily activities.

AR1004, known as *banhasasim‐*tang (BHS), is a natural herbal product used in traditional Korean medicine for the treatment of dyspepsia. In a lipopolysaccharide (LPS)‐induced mouse model, AR1004 was able to alleviate cognitive impairment and neuroinflammation. Moreover, in a scopolamine‐induced mouse model, AR1004 also demonstrated neuroprotective effects and improved cognitive function.

**Method:**

AriBio Co., Ltd. is conducting a Phase 2, placebo‐controlled trial for AR1004. The trial will take place in South Korea and will enroll up to 100 55‐80 years old males and females with MCI confirmed by cognitive assessment and plasma ptau‐217 levels. Participants will be randomly assigned in a 1:1 ratio to orally administer either AR1004 or matching placebo twice daily for 24 weeks. The primary endpoint is change from baseline in ADAS‐Cog 13. Secondary endpoints include Neurological Screening Battery tests of memory, attention, language, and executive function. Biomarker evaluations include Aβ42/40 ratio, GFAP, NfL, and pTau‐217. Safety will be assessed throughout the trial.

**Result:**

Results are expected to be reported in 2026.

**Conclusions:**

Traditional medicines such as AR1004 have been used extensively by the general public, but have rarely been tested in clinical trials of cognitive disorders. This Phase 2 trial will determine if AR1004 has benefit in improving cognitive and functional performance in people with MCI. IND approval has been granted by the Ministry of Food and Drug Safety (MFDS), and patient enrollment is planned to begin H2 2024. If results show that AR1004 has benefit in improving cognitive function, it will offer a safe alternative to the currently available treatment options.

